# The association between physical activity and hospital payments for acute admissions in the Australian population aged 45 and over

**DOI:** 10.1371/journal.pone.0218394

**Published:** 2019-06-24

**Authors:** Amir Marashi, Shima Ghassem Pour, Vincy Li, Chris Rissel, Federico Girosi

**Affiliations:** 1 Translational Health Research Institute, Western Sydney University, Sydney, NSW, Australia; 2 Capital Markets CRC, Sydney, NSW, Australia; 3 Digital Health CRC, Sydney, NSW, Australia; 4 Charles Perkins Centre, University of Sydney, Sydney, NSW, Australia; 5 Office of Preventive Health, NSW Ministry of Health, NSW, Australia; Dasman Diabetes Institute, KUWAIT

## Abstract

Physical activity (PA) is a key component of a healthy life, and it is hypothesised that individuals with higher levels of PA utilise fewer hospital resources. Quantifying the association between PA and hospital resource use is of interest to both payers and planners but estimates of its size in the general population are rare. In this paper we provide estimates of the association between PA and payments to hospitals in the Australian population over age 45. We use data from 45 and Up Study, a survey that contains health and lifestyle factors information about approximately 260,000 individuals over age 45 living in NSW, linked to hospital and death data. The linked data set allows to define a unique indicator for the level of PA over the week prior to the survey interview and to calculate payments to hospitals over the next year. We use Coarsened Exact Matching and multivariate analysis to study the relationship between PA and hospital payments, controlling for chronic health conditions, risk factors, standard socioeconomic variables and death. Our results clearly indicate that there is a statistically significant association between PA and lower hospital payments. While the size of the association depends to some extent on the covariates used in the model the conclusions are robust to changes in model specification. We also perform a sub-group analysis and show that the cost savings associated with PA are significantly larger for older and lower income populations. This study shows that if one is interested in lowering hospital expenditures then increasing PA levels is a policy that has the potential of being effective. It also shows that one does not need to target the entire population to achieve cost savings but can limit the intervention to the older population and/or the one in the lowest socioeconomic status.

## Introduction

In the last decade many developed countries have experienced rapidly rising rates of health spending, raising serious issues of sustainability, especially since three-quarters of health spending come from public funds [1]. In Australia, the total government health expenditure in 2015-16 was reported to be $114.6 billion, of which 40.9% was on public hospital services [2]. Health service providers and policy makers are interested in implementing interventions that maintain people in good health for longer periods, reduce the number of hospital admissions and reduce overall payments.

Since there is a well-established literature documenting the benefits of regular physical activity (PA) on some chronic health conditions and health-care expenditures, the NSW Office of Preventive Health has been considering policies aimed to increase levels of PA in the NSW population and reduce hospitalisations. In order to design such policies, it is crucial to have estimates of the size of the association between physical activity and hospital admission expenditures for the NSW population. Unfortunately, despite a large body of literature on this topic, such estimates are not available for NSW and they cannot be inferred from other studies. In fact, Sari [3] has published a review of the literature, up to year 2011, on the impact of physical activity on health-care utilisation on older adults, and found that while there is evidence that physical activity results in lower utilisation of health-care services the size of the effect varies significantly from study to study. A number of more recent studies support a similar conclusion [4–9]. Sari’s review shows that there is a variety of mechanisms through which increased PA can lead to reduction in health care utilisation. For example, in the hospital setting, there is evidence that PA reduces both the rate of hospitalisation and the length of stay, allowing for quicker recovery. However, there is also evidence supporting the hypothesis that PA reduces emergency admissions as well as the overall number of GP visits.

There are also several studies exploring the cost effectiveness of interventions promoting physical activity. Vijay et al. [10] have done a systematic review of these studies which mostly focus on brief interventions such as promoting pedometer use, suggesting exercise on prescription, motivational interviews or GP advice on exercise. There are other examples of studies focusing on specific interventions such as programs to encourage more active transport, free access to leisure centres, GP referral to an exercise physiologist and programs that encourage use of pedometers and mass media-based community campaigns [11–15]. Again, since these studies have been performed on different populations, using different variables and with different definitions of physical activity and payments they lead to very different results for the effect of physical activity. This implies that NSW health-care policy planners cannot simply rely on published numbers in the literature, but need to take advantage of data which are specific to the NSW population. From a methodological point of view this implies that this research question should be investigated by linking NSW-specific survey data, containing information about PA levels, risk factors, health status, demographics variables and socio-economic status, with administrative data, containing information about hospital use.

Policy makers also need information about what are the mechanisms leading from PA to reduced costs, and which particular chronic conditions should be targeted for intervention. For example, it is possible that PA reduces the incidence of heart disease but not hypertension. This information is clearly important and it is the subject of a separate study, focused on the relationship between PA and incidence of chronic conditions.

In this paper we use linked data to study the association between PA and hospital payments in the NSW population over age 45, independently of the health conditions affected by PA interventions. Our goal is to estimate the size of the association not only for this population but also for subgroups defined by age and income levels, so that the NSW Office of Preventive Health can more easily select target groups for interventions.

## Data and variables

### Data sets

This study uses data from the Sax Institute’s 45 and Up Study [16], a large cohort study of more than 260,000 New South Wales (NSW), Australia adult residents age over 45 years, recruited between 2006 to 2009. Prospective participants were randomly sampled from Department of Human Services (formerly Medicare Australia) enrolment database and people older than 80 years and those living in rural and remote areas were over sampled. The participants filled in a questionnaire and provided their data which included demographics, health conditions and life style behaviours and signed consent for follow-up and linkage of their information to routine health databases. Despite the overall low 18% response rate, the sample includes 11% of the targeted NSW population (i.e. adults 45 years and older). Mealing et al. [17] show that exposure-outcome relationship patterns derived from the 45 and Up Study are comparable with those derived from the New South Wales Population Health Survey.

The Centre for Health Record Linkage (CHeReL) [18] has linked the 45 and Up Study data with a range of administrative data sets including NSW Admitted Patient Data Collection (APDC) (which we refer to as hospital data) and NSW Registry of Births, Deaths and Marriages (RBDM). We use the hospital data to calculate one-year hospital payments for each person as explained in the next section. We also use the NSW Registry of Births, Deaths and Marriages (RBMD) to control for the death of the participants. The conduct of the 45 and Up Study was approved by the University of New South Wales Human Research Ethics Committee (HREC). Participants of the study gave consent for their data to be linked to any administrative data sets. Data from the study had been fully anonymised by the Sax Institute, who has administered the survey and acts as data custodian. Ethics approval for this study was granted by the NSW Population and Health Services Research Ethics Committee (reference: HREC/15/CIPHS/4) and the data were accessed using the Secure Unified Research Environment (SURE) [19].

### Variables

In this paper we investigate the relationship between hospital payments for acute admissions, the primary outcome, and *sufficient* level of physical activity, they key predictor. Since this relationship is also affected by a number of other factors, we include several covariates in the study. In this section we provide details about how all these variables were created.

#### Primary outcome: Hospital payments for acute admissions

The financial implications of higher levels of physical activity are of interest to a variety of stakeholders, with different stakeholders interested in different financial variables. For example, policy makers, acting as representative of the people, are often interested in cost savings to society as a whole, while hospital managers may be more interested in costs to hospitals. In Australia, State Governments are responsible for managing public hospitals and for funding a large component of the care they provide. Therefore, State Health Departments have strong interest, together with private insurers, in understanding how physical activity may impact payments to hospitals, and this is the perspective we take in this article. While other perspectives are equally interesting, they would not be supported by the data at hand. For example, if we wanted to take the perspective of the hospital we would need to know how much resources are used for each admission, something which is not recorded in the administrative data set available. However, the administrative data set at our disposal has enough information to estimate the total payment to the hospital.

Therefore, we choose as dependent variable the sum of payments for acute hospital admissions for each individual in the year following participation in the 45 and Up Study. Acute care, as defined by the Independent Hospital Pricing Authority (IHPA), is “care in which the primary clinical purpose or treatment goal is to: manage labour (obstetric), cure illness or provide definitive treatment of injury, perform surgery, relieve symptoms of illness or injury (excluding palliative care), reduce severity of an illness or injury, protect against exacerbation and/or complication of an illness and/or injury which could threaten life or normal function, perform diagnostic or therapeutic procedures and excludes care which meets the definition of mental health care” [20]. We only consider acute care admissions based on the acute care flag in the hospital data and we exclude the admissions related to Hemodialysis and Chemotherapy based on the Australian version of the Diagnosis Related Groups (DRG) codes, known as the Australian Refined DRG (AR-DRGs). We also remove items with total payments of more than $100,000 in order to exclude outliers from the analysis. This involves 435 people, which account for less than 0.16% of the data. The procedure used to estimate hospital payments is described below.

Since 2012, the Australian Government funds public hospitals across Australia through Activity Based Funding (ABF). The idea underlying ABF is that hospitals are reimbursed based on the expected value of cost of admission. This is done by assigning an Australian Refined-Diagnosis Related Groups (AR-DRG) code to each admission based on the diagnoses of the patient and the procedures that have been performed. AR-DRG codes have been designed in such a way that admissions with the same code utilise approximately the same amount of resources and have similar length of stay (LOS). Each AR-DRG is then assigned a price weight (PW), which is a relative measure of resource utilisation, and payments amounts are computed multiplying the price weight by the National Efficient Price (NEP). The NEP is updated and published each year by IHPA [21] and represents the average cost of an acute admission. This procedure does not account for the fact that some hospitalisations can be unusually long or short and in order to compensate the hospital appropriately some corrections based on the actual LOS are applied. Further small adjustments are also applied based on circumstances that may affect resource use and labour costs, such as time spent in the ICU and average local wages.

In our current data set we were able to perform the adjustments based on LOS, but did not have all the details required to apply the additional adjustments. This implies that we might be slightly underestimating the overall payments to hospitals, and we will discuss this in the concluding section. Since the National Efficient Price (NEP) was first published in year 2012, which is very close to the data collection years, and it is consistent with the Australian Refined-Diagnosis Related Groups (AR-DRG) version 6.0 used in the data, we estimated hospital payments using the NEP of 2012 and applied the Australian bureau of statistic consumer price index (ABS-CPI) conversion rate [22] to obtain 2018 AUD figures.

#### Key predictor: *Sufficient* physical activity

We define *sufficient* PA as having at least 150 minutes of Moderate to Vigorous Physical Activity (MVPA) and at least 5 sessions of physical activity in the past week, as suggested by the National Physical Activity Guidelines for Australians [23].

We derive Physical Activity (PA) data in the 45 and Up Study from questions 16 and 17 of the questionnaire. Question 16 asks participants “How many TIMES did you do each of these activities LAST WEEK?” and question 17 asks “If you add up all the time you spent doing each activity LAST WEEK, how much time did you spend ALTOGETHER doing each type of activity?” For both questions, the questionnaire describes the activities as below:

- Walking continuously, for at least 10 minutes (for recreation or exercise or to get to or from places)
- Vigorous physical activity (that made you breathe harder or puff and pant, like jogging, cycling, aerobics, competitive tennis, but not household chores or gardening)
- Moderate physical activity (like gentle swimming, social tennis, vigorous gardening, or work around the house)

We follow the guideline of The Active Australian Survey to define a single Moderate to Vigorous Physical Activity (MVPA) variable:

“Total time in minutes for each activity is calculated by multiplying the hours by 60 and adding the minutes. … To avoid errors due to over-reporting, any times greater than 840 minutes (14 hours) for a single activity type are re-coded to 840 minutes. Missing values are not imputed. Total time in activity overall is calculated by adding the time spent in walking and moderate activity and twice the time spent in vigorous activity. The time spent in vigorous activity is doubled because vigorous activity is more intense and so confers greater health benefits than moderate activity [24].”

An important issue with the survey data is dealing with missing values. Since the PA variable is the sum of all minutes and hours readings against PA for all three activities, absence of any of those values will result in a missing value for the PA variable. We perform the following pre-processing four steps to reduce the number of missing values and to correct improbable values that are highly likely to be mistakes:

1. For each activity, if the number of activities in the week is zero we set to zero both the hour and the minutes variables.
2. For each activity, if the number of activities in the week is missing and both the hours and the minutes are zero, we replace the missing value with zero.
3. For each activity, if one of the hours and minutes variables has a value and the other one is missing we replace the missing value with zero.
4. For each activity, if the number of minutes is zero and the average number of hours per session of activity is greater than 10, we assume that the hour has been mistaken with the minutes, so we divide it by 60.

Table 1 shows the prevalence of missing values for each of the 9 physical activity variables before and after the pre-processing step. After this pre-processing, 25.4% of items have missing values in the final *sufficient* PA variable (68,124 out of 267,897).

**Table 1:** Prevalence of missing values for PA variables before and after pre-processing (in percent).

	walk(%)			moderate(%)			vigorous(%)		
	num.	mins	hrs	num.	ins	hrs	num.	mins	hrs
raw	6.7	34	39	10	40	36	19	43	58
pre-processed	6	8	8	8	11	11	18	18	18

#### Covariates

The multivariate model used for the analysis, which is described in the next section, includes covariates such as sex, age, income, marital status, type of private health insurance (PHI) as well as risk factors such as history of regular smoking, body mass index (BMI), presence of four health chronic conditions (heart disease, hypertension, stroke, diabetes) and Physical Functioning Score. The model also includes a binary variable that takes the value of one if an individual died within one year of the survey completion date. While most of these variables are easily derived from the data, the physical functioning score is a combination of several survey questions and its construction is outlined below.

#### Physical functioning score

In the 45 and Up Study there is a question which asks participants whether their current health status limits them to perform some specific activities. This question is from the RAND Medical Outcome Study, 36-Item Short Form Survey Instrument (SF-36) [25]. The activities are:

- Vigorous activity (e.g. running, strenuous sports)
- Moderate activity (e.g. pushing a vacuum cleaner, playing golf)
- Lifting or carrying shopping
- Climbing several flights of stairs
- Climbing one flight of stairs
- Walking one kilometres
- Walking half a kilometre
- Walking 100 meters
- Bending, kneeling, or stooping
- Bathing or dressing yourself

The respondents can answer to these questions with three choices:

1. Yes, limited a lot (score: 0)
2. Yes, limits a little (score: 50)
3. No, not limited at all (score: 100)

The outcome variable from this question is a number between 0 and 100, which is the average score across all the items, with higher score defining a more favourable health state. This variable is highly correlated with physical activity and the effect size of PA on payments is sensitive to it.

## Methods

In this section we describe the methodological challenges we encountered while addressing our key research question and how we solved them.

### Re-weighting

The 45 and Up Study cohort has lower rates of smoking and higher rates of physical activity when compared to the NSW population, and it represents an overall younger and healthier population [17]. Therefore we apply the iterative proportional fitting (IPF) [26] method to re-weight the data and make it more representative of the NSW population. The IPF assigns different weights to different individuals in the data in order to reproduce the joint or marginal distributions of some targeted variables of a reference data set, which in our case is the NSW Population Health Survey of 2008. We decided to target the joint distribution of age groups and PA and marginal distribution of smoking, body mass index (BMI), and income.

### Matching

In this study we are interested in the association between PA and payments to hospitals so that we can estimate how those payments would change if we change the levels of physical activity in specific population groups. To answer these questions we need to use the observed data (factual outcome) and generalise it to situations that have not been observed (counterfactual outcome). Such generalisation requires a number of assumptions about the data in order to be valid. The main assumption is known as “Ignorability assumption” and assumes that given pre-treatment covariates *X*, treatment assignment is independent from the potential outcomes. Since our data is collected from an observational study, the assignment of the treatment (having *sufficient* physical activity) is not randomised and the two groups with *sufficient* PA (treatment) and *insufficient* PA (control) have systematic differences. Another assumption is called “positivity” and it implies that if a subset of the observed data only belongs to either control or treatment group, it cannot be used to calculate its counterfactual.

Matching is a technique that is used to prepare the data for the counterfactual analysis. It finds similar people from the control and the treatment groups and deletes the rest in order to get balanced groups of control and treatment. When the two groups are balanced the distribution of the covariates (*X*) are similar in the control and the treatment group. In this situation, as long as there are no unobserved covariates that correlate with both treatment and outcome, the difference between the average outcomes for two groups is equal to the causal effect and controlling for the *X* is not needed any more. However, in practice, the two groups are only approximately balanced and we still need to control for *X* using a statistical tool such as a regression model.

In this paper we use a matching method called Coarsened Exact Matching (CEM) [27]. CEM achieves a better uni-variate and multivariate balance of covariates for treatment and control groups and reduces the estimation bias more compared to other popular matching methods such as Propensity Score Matching (PSM) and Genetic Matching [28]. To apply CEM, the first step is to cut the continuous and ordinal variables into intervals and combine some of the levels of categorical variables. Then the algorithm matches treated units with control units falling in the same cells. The intervals of the continuous variables and the categories that are selected in the first step define the limits of these cells. Cells containing observations which are all controls or all treatments are removed. One can change the level of matching by changing the predefined intervals and categories.

CEM can perform a k-to-k or many-to-many match in each stratum. In the first case, for each treated unit in the stratum, a match from the control group can be selected at random or by a nearest neighbour algorithm. K-to-k matching generates two balanced groups with the same number of items in each group, which makes the further analysis easier. The downside of k-to-k matching is that it may ignore a large number of data points. In case of many-to-many matching the minority group units in each cell get a weight of one, while other units get weights that equalise the variance distribution in each cell across the treatment and control groups.

The imbalance between two groups could be measured in different ways. Iacus et al. [29] have suggested an intuitively appealing measure that is based on the *L*_1_ distance between the multivariate empirical distributions of the covariates in the treated and control groups. The outcome value is a number between 0 (two distributions overlay completely) and 1 (two distributions are completely different). We use the many-to-many version of the matching algorithm and match over most of the selected covariates for the study. Age and Physical Functioning Score are two continues variables of the study. We divide age into 5 year intervals and divide Physical Functioning Score into 5 categories with equal lengths. The other variables are already categorical and remained untouched, except for the 4 chronic health conditions that were replaced by a single total count variable.

### Model selection

If matching were perfect and it had used all the possible confounders, the analysis would consist of simply comparing the hospital payments in the matched groups with and without *sufficient* PA. However, this is not always the case and we want to investigate the effect of including additional covariates. Therefore, we apply multivariate regression models to the matched data set. In particular we are interested in understanding the effect of controlling for death of the participants, which seems an important covariate, and we will explore regression with and without this variable.

Our selected regression model for the analysis of the association between PA and hospital payments is weighted linear regression. Model selection for highly skewed outcomes such as health-care expenditure has always been a much-debated topic in the literature. Violation of linear regression assumptions and heteroskedasticity [30] issues of expenditure data on one hand and re-transformation problem of logged data on the other hand has been discussed thoroughly in the field of econometrics [31–40]. These discussions are outside the scope of our study since our focus is not on individual level predictions but rather on the association between PA and payments. A previous study on the same data set, which used similar dependent variables, showed that the linear model produces the best fit compared to alternatives such as log transformed models, GLM models and two-part models [41]. Therefore, we used weighted linear regression estimated by ordinary least square (OLS) for simplicity of interpretation and in order to avoid the re-transformation issues.

## Results

The dataset consists of 267,897 records, out of which 199,773 allow for the computation of the *sufficient* PA variable. We imputed BMI, marital status and household income using Multinomial Log-linear Models from the “nnet” R package [42] and removed items with missing value in the other relevant covariates. We also exclude 206 participants with annual payments more than $100,000 ending up with 178,755 individuals for the analysis. Nearly 70.4 percent of males and 72.5 percent of females have reported *sufficient* physical activity, which is higher than the 47.6 percent reported statistics in the NSW Population Health Survey [43]. After re-weighting, overall *sufficient* PA is reduced to 49.7%.

Table 2 shows the variables of the study for the groups of participants with *sufficient* and *insufficient* PA, after re-weighting for different covariates. In general, the physically active cohort are younger and in better health, and the *L*_1_ measure of imbalance before matching is 0.363. The matching algorithm removes 11,701 (9%) participants from the active group and 6,488 (13%) participants from the group with *insufficient* PA, reducing the *L*_1_ measure to 0.

**Table 2:** Comparison of the variables of the study for two groups of people with *sufficient* PA and *insufficient* PA.

Variable	Insufficient PA	Sufficient PA
**n**	48969	129786
**Sex = M (%)**	54.4	50.5
**Age category (%)**		
45-54	21.8	28.3
55-64	28.7	34.7
65,74	24.7	23.7
75-79	19.0	11.7
≥80	5.8	1.6
**Marital status (%)**		
partnered	73.7	78.5
separated	10.3	9.9
single	5.1	5.1
widowed	10.9	6.5
**BMI (%)**		
normal	31.4	40.3
obese	28.3	17.9
overweight	38.3	40.4
underweight	1.9	1.3
**Household income (%)**		
<20K	25.6	15.7
20K-50K	34.2	33.3
50K-70K	14.3	16.0
≥70K	25.9	35.1
**Ever smoked regularly (%)**	56.7	54.2
**Hypertension (%)**	42.0	34.7
**Heart (%)**	17.6	11.8
**Stroke (%)**	5.7	2.3
**Diabetes (%)**	13.0	7.2
**Num. chronic health conditions (%)**		
0	45.7	57.0
1	35.1	32.0
2	14.8	9.3
3	3.8	1.5
4	0.5	0.2
**Private health insurance (%)**		
none	14.9	14.6
DVA	3.3	1.9
extra	40.9	48.9
healthcare card	30.4	22.5
no extra	10.5	12.1
**Physical Functioning Score (mean (sd))**	69.06 (31.87)	87.18 (18.44)
**Died in the next year (%)**	3.2	0.7
**Cost (mean (sd))**	4100.35 (10234.94)	2218.39 (6826.64)

After re-weighting, 28.4% of our selected data have at least one record of acute type hospital admission in the hospital dataset in next year. The average hospital payment in this population is about $11,111 (95% CI = 10,944 to 11,277) with median of $6554 (95% CI = 6,439 to 6,674), while the average payment over the whole population, with and without admissions, is $3,164 (95% CI = 3,106 to 3,221).

Prior to matching, the average difference in payments for people with *insufficient* PA and *sufficient* PA is $1,882 (95% CI = 1,768 to 1,996) with some of the difference due to the different characteristics of the two groups. After the matching, the weighted average difference reduces to $477.6 (95% CI = 393.2 to 562.0). If matching were perfect this would be our final estimate, but as explained in the methods section it is important to investigate the effect of including additional covariates, and in particular death of the participant. Therefore we report in Table 3 the results of two linear regressions, with and without the death covariate, and for both regressions we report the results with and without matching.

**Table 3:** The coefficients of the least square regressions.

	Model 1		Model 2	
	Without Matching	After Matching	Without Matching	After Matching
(Intercept)	6113.2(122.9)***	5563.1(130.8)***	5126.1(120.7)***	4824.9(128.2)***
**sex = M**	697.2(42.7)***	703.4(45.4)***	585.0(41.8)***	557.64(44.1)***
**age category**				
51-55	104.2(79.2)	105.1(83.9)	100.1(77.5)	111.8(82.0)
56-60	236.4(75.3)**	193.1(78.9)*	244.9(73.7)***	227.5(77.1)**
61-65	528.9(81.1)***	483.4(86.3)***	545.0(79.3)***	535.0(84.4)***
66-70	1330.1(83.1)***	1330.1(87.3)***	1289.7(81.3)***	1347.3(85.3)***
71-75	1722.5(94.3)***	1699.9(99.5)***	1691.1(92.2)***	1698.8(97.3)***
76-80	2154.3(102.8)***	2572.7(108.9)***	2018.9(100.5)***	2428.3(106.5)***
81-85	2417.(103.9)***	2436.3(108.1)***	2049.8(101.7)***	2179.6(105.7)***
86-90	2644.1(149.0)***	2360.9(169.4)***	1966.0(145.8)***	1659.6(165.8)***
≥90	2352.6(223.6)***	1793.8(295.0)***	221.3(220.0)	264.7(289.0)
**marital status**				
separated	105.5(67.8)	141.6(69.4)*	122.9(66.3).	148.3(67.9)*
single	−53.3(91.1)	86.0(94.5)	−11.9(89.1)	104.8(92.4)
widowed	56.3(79.3)	−7.36(82.2)	70.1(77.6)	−25.9(80.4)
**household income**				
20K-50K	97.0(59.2)	135.9(61.0)*	116.8(57.9)*	180.4(59.6)**
50K-70K	314.4(76.0)***	300.3(83.3)***	319.5(74.4)***	324.2(81.4)***
≥70K	220.4(73.1)**	229.7(79.5)**	217.6(71.5)**	259.3(77.7)***
**stroke**	543.7(104.3)***	180.3(117.7)	394.6(102.0)***	36.0(115.1)
**heart**	1188.8(59.7)***	1177.2(62.8)***	1104.1(58.4)***	1062.8(61.4)***
**hypertension**	−3.3(43.2)	60.3(44.9)	77.2(42.3).	86.6(43.9)*
**diabetes**	600.2(68.4)***	521.7(71.3)***	520.7(66.9)***	371.4(69.7)***
**ever smoked regularly**	272.6(40.8)***	359.2(43.2)***	209.6(39.9)***	269.1(42.2)***
**Private Health Insurance**				
DVA	1577.5(139.7)***	1310(179.1)***	1642.2(136.6)***	1079.1(175.2)***
extra	761.8(61.9)***	733.4(66.0)***	751.1(60.5)***	710.2(64.6)***
healthcare card	453.3(69.1)***	327.6(73.8)***	440.1(67.6)***	272.3(72.1)***
no extra	360.2(79.2)***	332.6(87.3)***	395.7(77.5)***	338.2(85.3)***
**BMI**				
obese	−257.5(56.0)***	−7.9(57.5)	−87.1(54.8)	152.9(56.2)**
overweight	−81.2(46.6).	76.1(50.5)	15.1(45.6)	180.5(49.4)***
underweight	233.5(160.1)	445.6(245.9).	−203.3(156.6)	306.1(240.5)
**Physical Functioning Score**	−65.5(0.9)***	−60.8(0.9)***	−55.3(0.9)***	−53.3(0.9)***
**died in the next year**			12982.3(144.1)***	13779.3(160.7)***
**sufficient PA**	−325.4(42.1)***	−426.8(41.4)***	−259.8(41.2)***	−327.8(40.5)***
*R*^2^	0.09	0.08	0.13	0.12

*** *p* < 0.001,

** *p* < 0.01,

* *p* < 0.05

Our preferred specification includes the death covariate, since we know that hospitalisation usage is much higher in the last year of life [44–46]. The analysis shows that in this case *sufficient* physical activity on average reduces the annual hospital admissions payments by $327.7 (95% CI = 248.4 to 407.2). If death is not included this number climbs to $426.8 (95% CI = 345.7 to 508.0). Table 3 also shows the coefficient for Model 1 and 2 on unmatched data. In both models, the coefficient of PA is smaller compared to the matched models.

**Fig 1:**
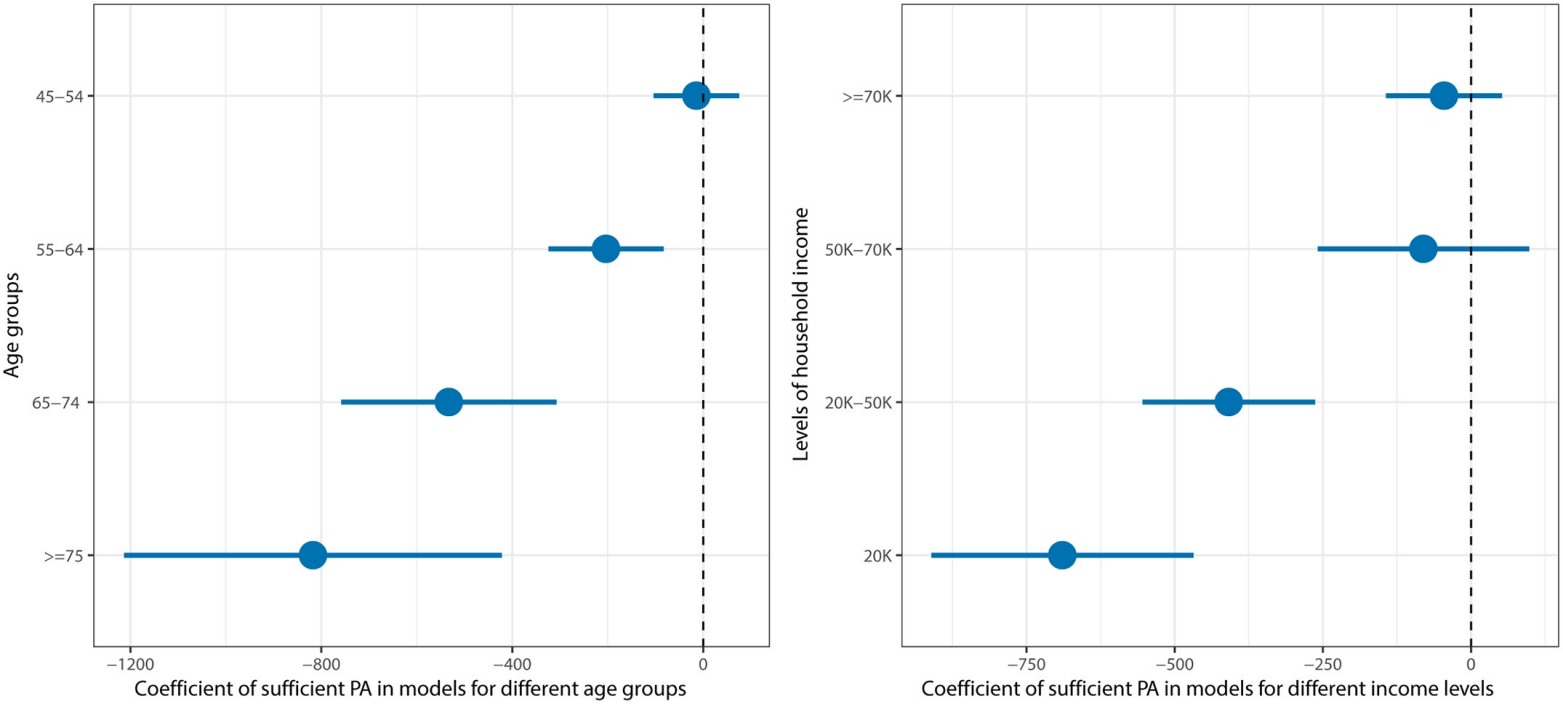
The coefficient of *sufficient* PA in the regressions for different sub-populations. (left): Coefficient of *sufficient* PA in models for different age groups, (right): Coefficient of *sufficient* PA in models for different income levels.

We also performed subgroup analysis, since the results of (Table 3) apply to the average participant. In particular we are interested in understanding how the effect of *sufficient* PA varies with key variables such as age and income. The results of the analysis for separate age groups and separate income levels are presented in Fig 1. The figure shows that the effect of *sufficient* physical activity is statistically significant for most of the age groups and for the two lowest income groups. The effect size for the oldest group is $817.56, which is much higher than the effect for the youngest groups. The analysis based on the household income also shows that the effect size differs considerably based on income. The potential saving associated with *sufficient* PA is more than 15 times bigger for the cohort whose income is less than 20 thousand dollars per year compared to the cohort with more than 70 thousand dollars annual income.

## Sensitivity analysis

There are few items that we considered in our sensitivity analysis:

- In our original analysis we also included in the regression specification education level and psychological distress scale (K10). However, excluding these variables did not change the effect of PA nor affected any of the conclusions and therefore we have not included these variables in Table 3.
- About 50% of all hospital admission data linked with the 45 and Up Study is from private hospitals, which may or may not use the Australian Refined-Diagnosis Related Groups (AR-DRG) payment system. However, it is known that public and private hospitals have similar average costs [47]. Since we are interested in understanding the effect of PA on overall hospital costs, independently on whether admissions were into private or pubic hospitals, we kept both private and public admissions in the data. If we limited the analysis to public admissions the effect of *sufficient* PA would be somewhat smaller and equal to a saving of $268.2 (95% CI = 209.8 to 326.6).
- As common in this type of analysis, we have tried a variety of choices for the matching variables. For example, we excluded BMI and Physical Functioning Score from the matching and studied the effect of these variables using multivariate regression. We also tried different levels of coarseness for the age and the Physical Functioning Score variables. In all cases we have obtained estimates of the effect of *sufficient* PA on hospital payment which were consistent with the ones reported in Table 3. Therefore our overall assessment is that the estimate provided in this paper is quite robust to the specification of both matching and regression.
- The Activity Based Funding (ABF) payment system takes in account the fact that individuals of Aboriginal and Torres Strait Islander origin are associated with costs that are 10% higher. In our data we do not have a variable that allows to identify this population and are unable to perform this adjustment. However, given the very small size of this population this omission is unlikely to affect the results in any significant way.

## Discussion

In this study we have investigated the relation between *sufficient* physical activity and acute hospital payments for Australians aged 45 years and older in the state of NSW. While this relationship has been studied in the past, this is the first study that produces a quantitative estimate of this effect in Australia. The study uses data from the 45 and Up Study survey of the NSW population, which is not necessarily representative, but we have used the IPF [26] to re-weight the data in such a way that it becomes more representative of the NSW population, and we have applied matching techniques [27] to overcome some of the limitations of observational studies and make the results less sensitive to functional form specifications.

We have found that on average, having *sufficient* physical activity reduces hospital payments of 327.8 dollars a year per person. One important finding is that the size of the effect is different for different sub-groups of the population and the potential savings in health-care payments is bigger in the population with lowest household income. This is likely to be related to the fact that in NSW, as in many developed economies, the low-income population has worse health status and a higher number of chronic conditions than average [48], while the higher income population enjoys better health. But if the higher income population is healthier than average it is unlikely that PA could have much effect on expenditures, and therefore the effect of PA on expenditures has to grow when we limit the analysis to the low-income population, as we have observed. A similar rationale applies to the older age groups, who have more chronic conditions and are in worse health than average, and it is corroborated from our finding that the effect of PA on hospital expenditures is higher in the oldest age group.

These results are in line with the general expectation that physical activity is associated with reductions in health-care costs and is more beneficial to the least healthy populations. The reduction in hospital costs could be due to a variety of reasons: fewer hospital admissions, shorter length of stay, or a different distribution of AR-DRGs, with smaller cost weights due to better general health status and fewer complications. The results regarding the variation of the effect size across age groups complement the findings of Khoo et al. [49] which show that older groups are the highest consumers of hospital resources and should be targeted for physical activity interventions.

The results of this paper have clear practical implications on the design of preventive strategies, which we now outline. At the end of 2017 the size of the NSW population was more than 7.8 million, out of which 3.17 million people (41%) were older than 45 years [50], with a reported rate of *sufficient* PA equal to 48.5% [51]. If we could design an ideal awareness campaign that brings the rate of *sufficient* PA in this population to 100%, applying the estimated effect size of $327.8 per person, we find that the potential savings on hospital payments is 535 million dollars per year. Such a campaign would be expensive, because it would target the large and heterogeneous population over age 45, and would require to design different strategies for different subsets of the population. The campaign would also be inefficient, since we have shown in the previous section that the benefits of *sufficient* PA are unequally distributed across the population and therefore it would target many individuals associated with little benefit.

This paper suggests that we could achieve significant savings by designing more targeted and less complex campaigns that focused on more homogeneous groups. For example, we could target the 552,000 individuals over age 75 who are associated with a much higher benefit (817 dollar per person, that is 2.5 times larger than the one in the baseline population over age 45). This population has also lower level of *sufficient* PA, relative to baseline (35.5%, compared to 48.5%, [50, 51]) and therefore there is relatively more people to target. As a result, the potential savings of such a campaign are equal to 291 million dollars per year. This is 54% of the savings of a full campaign, but this approach corresponds to a much more efficient strategy since it targets only 17.4% of the baseline population and it requires a less complex design.

Alternatively one could target a segment of the population defined by low income or concession status. In fact, if we focused on those living in households with income less than $20,000 we would obtain figures and conclusions very similar to those obtained for the population over age 75.

The numbers we have reported are conservative and are likely to underestimate the size of the effect of PA for two main reasons. The first is that we have only focused on acute care and have not considered the potential effect on other types of care such as ED admissions, medications, physicians visits, allied health and residential aged care. The second reason is that our calculated payments do not take in account all the adjustments that are usually made in the Activity Based Funding (ABF) payment system, which would mostly increase the costs.

We have applied a matching technique to balance the treatment and control groups in term of the known confounding variables between PA and hospital payments and make the results more robust against functional form specification. While we have experimented and controlled for a rich set of potential confounders, in every observational study there could always be some unobserved confounder, and therefore we cannot conclusively assert that reported effect sizes in the study have a causal interpretation. In general, though, our estimates are in line with what has been reported in the literature worldwide [4, 5, 52].
